# Use of Plasmapheresis in Managing the Diagnostic Dilemma of Symptomatic Hypertriglyceridemia

**DOI:** 10.1155/2012/501373

**Published:** 2012-12-05

**Authors:** Nigel Gordon Maher, Hariharan Ramaswamykanive

**Affiliations:** ^1^Department of Medicine, Manning Base Hospital, Taree, NSW 2430, Australia; ^2^Department of Intensive Care, Manning Base Hospital, Taree, NSW 2430, Australia

## Abstract

We present a case study of a 29-year-old male who presented with abdominal pain typical for pancreatitis. Detailed history and investigations revealed that the cause of abdominal pain was secondary to the raised triglyceride levels. It was difficult to distinguish whether he had hypertriglyceridemia-induced abdominal pain or acute pancreatitis, given that he had only a mildly raised lipase and a normal contrast computed tomography scan of the pancreas. The abdominal pain resolved with the fall in the triglyceride levels following plasmapheresis. Plasmapheresis is an underevaluated modality of the treatment of hypertriglyceridemia due to its cost and availability.

## 1. Introduction

Severe hypertriglyceridemia is known to cause abdominal pain and acute pancreatitis [1]. Amylase and lipase serum levels, both diagnostic markers for pancreatitis [2], may be spuriously normal or only mildly raised in the setting of hypertriglyceridemia [3–5], obscuring a diagnosis of pancreatitis.

Management of hypertriglyceridemia is generally conservative, such as dietary advice, fasting, lipid lowering medications, and noncaloric intravenous fluids [1, 3]. Plasmapheresis has emerged as an effective, yet underevaluated, technique for rapidly lowering triglyceride levels, helping to prevent and treat acute pancreatitis [6, 7]. No reports have documented its use in the symptomatic management of abdominal pain, independent of pancreatitis, induced by severe hypertriglyceridemia.

## 2. Case Report

A 29-year-old indigenous Australian male presented to the emergency department of a rural hospital complaining upper abdominal pain radiating to the back. His heart rate was 64 beats per minute, blood pressure 138/83 mmHg, respiratory rate 11 per minute, saturating 99% on room air, and he had a temperature of 36.5°C. He had 8 out of 10 on visual analogue pain score (VAS). His past medical history included asthma, type II diabetes, obesity, and hypertriglyceridemia.

He was diagnosed to have hypertriglyceridemia-induced pancreatitis three years ago when he presented with severe abdominal pain. His triglyceride level then was 39 mmol/L. The computed tomography (CT) scan at that time confirmed a diagnosis of focal pancreatitis, along with hepatosplenomegaly. He was lost to further follow-up, postconservative management. He was a current smoker and denied recent alcohol consumption.

**Figure 1:**
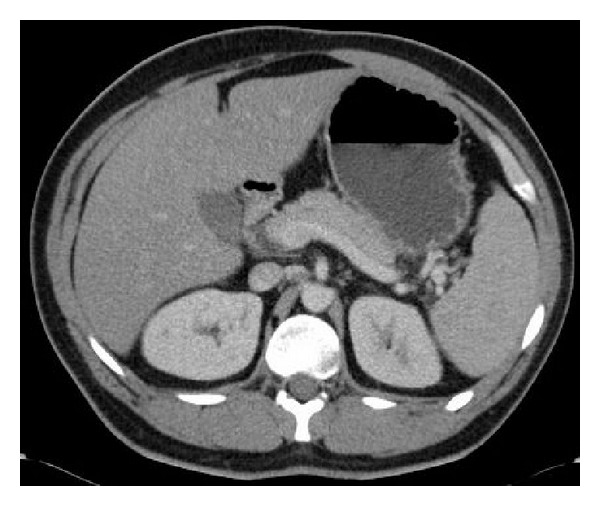
A coronal section of the contrast computed tomography scan, revealing a fatty liver and normal pancreas.

At admission he was clinically very dry. His blood tests were not fully analysable due to excessive lipemia. He was resuscitated with three litres of crystalloid fluid and on repeat blood testing, he was found to have triglycerides of 24.1 mmol/L (<1.70 mmol/L); cholesterol 8.4 mmol/L (<5.5 mmol/L); lipase 119 U/L (8–78 U/L). His estimated hematocrit and hemoglobin were high suggesting hemoconcentration. The white blood cell count was 12.3 × 10^9^/L (4–12 × 10^9^/L), corrected calcium was 2.29 mmol/L (2.22–2.53 mmol/L), and creatinine was 68 *μ*mol/L (63–111 *μ*mol/L). Random blood glucose testing indicated 21.3 mmol/L. Abdominal contrast CT done 24 hours after symptoms commenced revealed a fatty liver (Figure 1). There was no ketonuria. Electrocardiogram and troponin were normal.

As he did not have imaging features of pancreatitis or a significantly raised lipase level (more than twice the upper limit of normal), it was considered the abdominal pain may be due to a “hyperlipemic abdominal crisis” [1], although pancreatitis could not be ruled out. Nonetheless, his acute abdominal pain was attributed to hypertriglyceridemia. He was kept nil by mouth, administered intravenous crystalloid fluids, heparin, pantoprazole, thiamine and an insulin infusion. His triglyceride levels remained high on day 2 (20.7 mmol/L).

Despite aggressive fluid resuscitation and adequate analgesia, he continued to have abdominal pain and hence he was transferred to a tertiary centre for plasmapheresis. With 2 treatments of plasmapheresis over successive days, his triglyceride level reduced to 7.4 mmol/L. With that decrease in triglycerides, his abdominal pain resolved. He was discharged home with fenofibrate, atorvastatin, and metformin, and he remained well 4 weeks after admission.

## 3. Discussion

The cause for high serum triglycerides can be either genetic or acquired in origin. Acquired causes include uncontrolled diabetes mellitus, alcohol abuse, pregnancy, medications, hypothyroidism, nephrotic syndrome, and high carbohydrate diets [5, 6, 8, 9].

Complications of severe hypertriglyceridemia (>22.6 mmol/L) can result in acute pancreatitis, chronic or acute abdominal pain (the “hyperlipemic abdominal crisis”), hepatosplenomegaly, eruptive xanthomas, lipemia retinalis, peripheral neuropathy, memory loss/dementia, and dyspnoea [1, 3, 8]. The threshold for inducing pancreatitis may be at lower levels (>11.3 mmol/L) [9]. There are several proposed mechanisms underlying the pathogenesis in hypertriglyceridemia-induced pancreatitis. The first is that the raised levels of free fatty acids and lysolecithin released by pancreatic lipase activity are toxic to the pancreatic cells [4, 8]. The second is that in acidic environments, the free fatty acids can activate trypsinogen which then damages the pancreatic endothelium [4]. Furthermore, increased chylomicrons contribute to hyperviscosity within the pancreatic capillaries, which may lead to ischemia [9]. The mechanisms behind the abdominal pain are less clear. It may be due to serositis or the rapid hepatosplenomegaly that can develop with hypertriglyceridemia [1].

While pancreatitis is usually diagnosed from typical clinical symptoms and raised pancreatic enzymes that are at least twice the upper normal limit [2], in hypertriglyceridemia, these enzymes may be spuriously normal or only mildly raised [3–5, 10]. These effects have been well documented for amylase, but there is limited data regarding lipase levels. Lipase is a more sensitive and specific marker for acute pancreatitis compared to amylase [11], and lipase levels may be significantly raised (more than three times the normal limit) while amylase levels are normal in the setting of hypertriglyceridemia [12]. Furthermore, one study of hypertriglyceridemia-induced pancreatitis showed that all 9 patients who had both lipase and amylase measured recorded lipase levels at least four times higher than amylase levels [13]. This indicates that lipase analysis may be less affected by hypertriglyceridemia compared to amylase and is the preferred diagnostic choice in hypertriglyceridemia-induced pancreatitis. Another consideration is serial dilution of the lactescent serum to avoid triglyceride interference while running the assay, which may unmask an abnormally high amylase [10].

A recent review suggests, relying on clinical symptoms, a lipase level greater than three times the upper limit and imaging to support a diagnosis of hypertriglyceridemia induced pancreatitis [9]. Indeed imaging, usually via an ultrasonogram or CT, may aid diagnosis and help rule out other important differential diagnoses for abdominal pain (such as cholecystitis, appendicitis, peptic ulcer disease, and splenic or hepatic infarction [1]).

While efforts to confirm acute pancreatitis are prudent, it is worthwhile also considering that hypertriglyceridemia can mimic acute pancreatitis in its presentation, the so-called “hyperlipemic abdominal crisis” [1]. Features in support of this diagnosis in our patient were the severe hypertriglyceridemia (greater than 22.6 mmol/L), previously noted hepatosplenomegaly, normal contrast CT of the pancreas on this admission, and normal corrected calcium on admission. Conversely, features in favour of pancreatitis were the mildly raised white cell count and previous history of pancreatitis. A lipase level just over the normal limit was ambivalent in this situation.

Despite the diagnostic dilemma, in such clinical state the aim is to reduce endogenous and exogenous sources of triglycerides and promote their clearance. Exogenous sources can be reduced by low-fat diets and fasting; endogenous sources can be reduced by lipid lowering medications and limited alcohol intake [1, 3]; clearance can be promoted by insulin and heparin enhancement of lipoprotein lipase activity [8, 9], although heparin use remains controversial [9].

Plasmapheresis has emerged as an effective modality in rapidly reducing serum triglyceride levels and helping to treat and prevent hypertriglyceridemia-induced pancreatitis [6, 7]. Although plasmapheresis as a treatment for hypertriglyceridemia was first described in 1978 by Betteridge et al. [14], it remains poorly evaluated in the literature, most likely due to its cost and accessibility [9]. No randomised controlled trials in the English literature have been published on this specific topic. The American Society of Apheresis guidelines only refer to plasmapheresis for hypertriglyceridemia in the context of pancreatitis, for which it is given a category III strength of evidence [8], implying a “suggestion of benefit” [15].

Plasmapheresis involves separating the plasma from the blood using a membrane for filtration, and replacing it with either isovolumetric albumin solution or fresh frozen plasma [16, 17]. It requires central vein or dialysis catheter access and is performed under transient anticoagulation [9, 17]. Risks of plasmapheresis include infection, allergic reaction, and bleeding [9]. It has been shown to reduce triglyceride levels by an average of 61% after 1 session [6] and provide rapid resolution of pancreatitis symptoms [6, 7].

While conservative treatments have been effective in managing abdominal pain caused by hypertriglyceridemia [1], we illustrate that plasmapheresis can be effectively used to alleviate these symptoms particularly in patients, like ours, who may be experiencing a “hyperlipemic abdominal crisis,” have had covert pancreatitis, seem at imminent risk of developing fulminant pancreatitis, or who are noncompliant with conservative management [18]. The timing of plasmapheresis in managing hypertriglyceridemia remains unevaluated. In the context of hypertriglyceridemia-induced pancreatitis, early intervention, at least within 48 hours, is likely to be important and of benefit [7, 9, 17].

In conclusion, this paper highlights the potential benefit of plasmapheresis in resolving acute abdominal pain secondary to hypertriglyceridemia in patients who are noncompliant and are at risk of developing fulminant pancreatitis. Clinicians should be alert to the alterations in biochemistry caused by hypertriglyceridemia that may affect pancreatitis diagnosis. More research is needed to clarify the sensitivity and specificity of lipase in the setting of hypertriglyceridemia. Further study is also required to ascertain the efficacy, timing, cost-benefit ratio, and necessity of plasmapheresis in the setting of hypertriglyceridemia-induced abdominal pain where the definitive diagnosis remains unclear.
